# Acute stress show great influences on liver function and the expression of hepatic genes associated with lipid metabolism in rats

**DOI:** 10.1186/1476-511X-12-118

**Published:** 2013-07-31

**Authors:** Xiaoling Gao, Yuaner Zeng, Shuqiang Liu, Shuling Wang

**Affiliations:** 1 School of Chinese Pharmaceutical Science, Guangzhou University of Chinese Medicine, Guangzhou, Guangdong, China

**Keywords:** Acute stress, Lipid metabolism, Raged impairing liver, Scavenger receptor BI, ATP-binding cassette transporter

## Abstract

**Background:**

The theory of Chinese medicine believes rage harms normal liver function, namely ’raged impairing liver' in short. The purpose of this study is to investigate the impact of acute stress on liver lipid metabolism in rats.

**Methods and results:**

Comparison of liver function indicators, serum lipid level of rats under acute stress and normal rats, as well as detection of liver tissue in the SR - BI, ABCG5 and ABCG8 protein and gene expression changes. Acute stressed rats had shown a lower serum levels of albumin (*P*<0.01), HDL- cholesterol (*P*<0.01) than normal rats, with higher serum levels of globulin (*P*<0.01) and LDL-cholesterol (*P*<0.05). Acute stressed rat’s liver tissue exhibited a lower protein expression of ABCG5 (*P*<0.05), ABCG8 (*P*<0.01) and a higher level of SR-BI (*P*<0.05), compared with to normal rats. Furthermore, liver gene expression of ABCG5 (*P*<0.01) and ABCG8 (*P*<0.05) were lower in acute stressed rats than in normal rats, while SR-BI was higher in acute stressed rats than in normal rats (*P*<0.01).

**Conclusions:**

Acute stress had a direct influence on rat’s liver lipid metabolism.

## Background

The word ‘stress’ first appeared in the index of Psychological Abstracts in 1944 [1]. Recently, stress has shown to play an important role in the onset of cardiovascular diseases, immunological disorders and pathophysiological consequences of normal aging [2-4]. Stress had also been shown that will inhibit metabolic function, such as lipid elimination and glycometabolism [5,6]. The theory Chinese Medicine believes rage harms liver function [7]. Meanwhile, acute stress causes rage, so acute stress may individually damages the liver. There are reports [8-11] that acute stress increases oxidation and diminishes antioxidant’s protection, stating acute stress oxidates and destroys the liver. The liver is a crucial organ that facilitate lipid metabolism, including cholesterol metabolism and it is meaningful to study acute stress’s effect on lipid metabolism in rats. In recent years, the study of lipid metabolism focused on reverse cholesterol transport (RCT), which means excess cholesterol transport from peripheral cells of apolipoprotein to the liver by high density lipoprotein (HDL), where lipid uptake took place and cleared. ATP-binding cassette transporter (ABC) G5, G8 and scavenger receptor- BI (SR-BI) are principal mediators of cholesterol efflux [12-14]. ABCG5 and ABCG8 mediate cholesterol outflow from peripheral cells, while SR-BI mediate hepatic selective uptake of cholesterol esters. Present study aims to discuss the effect of acute stress on lipid metabolism in rats from the perspective of reverse cholesterol transport.

## Materials and methods

### Animals

Adult male Sprague Dawley rats weighing 160-180 g were obtained from the Central Animal Facility, Guangzhou University of Chinese Medicine (SCXK (Guangdong) 2008–0020), the rats were housed individually per cage upon arrival, they were allowed to adjust to their new environment in 1 week. The rat had free access to water and food, temperature of 25 ± 1°C, a 12-hour light–dark cycle (lights on at 08:00–20:00). Protocols were conducted in accordance with standard ethical guidelines, it was approved by the institution animal ethics committee (Grants 81102530/H2702 from National Natural Science Foundation of China). The rats were randomly selected into normal group and acute stress group (n = 8 in each group). Rats in the acute stress group were subjected to tail clipping at one third towards the end of the tail, three times a day for 30 minutes each time [15]. The normal control group were housed in a separate room and were not subjected to any stress for 14 consecutive days. Collection of blood, sacrifice of rats and liver isolation were performed after the restraint stress procedure.

### Sucrose consumption test

All rats were given 1% sucrose solution for a 24 hour period in their cages with no food and water following food and water deprivation for 24 hours. Sucrose consumption was recorded by re-weighing bottles of solution.

### Open-field test

Open-field test was conducted as described by Dunn [16], to assess acute stress effects on rats, the test was performed on the last 2 days before the end of the experiment. The apparatus consists of a rectangular box (80 × 80 × 50 cm), a floor divided into 25 (16 × 16 cm) rectangular units. The rats were placed in the center of an open field and were allowed to freely explore the area for 3 minutes. Two motor parameters were quantified throughout this test: locomotion frequency (numbers of with which the rat crossed one of the grid lines with all four paws) and rearing frequency (times of rearing with hind legs). The animals were acclimated to the experimental room for at least 2 hours prior to the beginning of the open-field test. Open-field tests were carried out in soundproof rooms without any human interference. The open-field was cleaned with a 5% water-ethanol solution before behavioral testing to eliminate bias due to odors left by previous rats. The evaluation of rat’s motivation behavior was performed in a blinded manner by two independent observers. After testing inter-observer reliability, the mean from the results was statistically analyzed.

### Biochemical analysis

Blood samples were collected under chloral hydrate anaesthesia and centrifuged at 3500 rpm for 15 minutes, serum was separated at 4°C by a refrigerated centrifuge (HEMA, China). Serum concentrations of total cholesterol (TC), total triglyceride (TG), low-density lipoprotein cholesterol (LDL-C), high-density lipoprotein cholesterol (HDL-C), alanine aminotransferase (ALT), aspartate aminotransferase (AST), albumin (ALB) and globulin (GLB) were measured by enzymatic assays using an automated biochemical analyzer (Cobas 8000, Roche, Germany).

### Quantitative real-time PCR

Total RNA was extracted with Trizol reagent (Invitrogen, Shanghai, China). The quality of samples was confirmed by agarose gel electrophoresis. Total RNA was reverse transcribed to complementary DNA using Prime Script® 1st Strand cDNA Synthesis Kit (TAKARA, Kyoto, Japan). The thermo cycler settings were 30°C for 10 minutes, 42°C for 60 minutes, and 70°C for 15 minutes. Real-time PCR was performed in triplicates using SYBR® Premix Ex Taq kit (TAKARA) in a 25 μL reaction volume. GAPDH was used as the housekeeping gene. The primers used for real-time PCR were listed in Table 1. Relative mRNA levels were calculated by the method of 2^-^△△^Ct^.

**Table 1:** Primers used for real-time PCR analysis

**Gene**	**Primer**	**Sequence(5′ → 3′)**
SR-BI	Sense	CCCCATGAACTGTTCCGTGA
	Anti-sense	CCACAGCAATGGCAGGACTA
ABCG5	Sense	GGGAAGTGTTTGTGAACGGC
	Anti-sense	GTGTATCTCAGCGTCTCCCG
ABCG8	Sense	ACGTGGACTTGACGAGCATT
	Anti-sense	GTGTCCTGTGTGAGGGTCTG
GAPDH	Sense	TGCTGGGGCTGGCATTGCTCTC
	Anti-sense	ATGAGGTCCACCACCCTGTTGC

### Western blot analysis

Tissue samples were frozen in liquid nitrogen. Total protein was isolated in a lysis buffer, resolved by 10% odium dodecyl sulfate polyacrylamide gel electrophoresis (SDS-PAGE) and transferred onto polyvinylidene fluoride (PVDF) membranes by electroblotting. SR-BI, ABCG5 and ABCG8 proteins were detected using an anti- SR-BI monoclonal antibody (1:500 dilution, Abcam, UK), anti-ABCG5 polyclonal antibody (1:500 dilution, Bioss, Beijing, China), or anti-ABCG8 polyclonal antibody (1:500 dilution, Bioss, Beijing, China). The bands were visualized with an enhanced chemiluminescence kit (Millipore, Billerica, MA) and analyzed with Image-Pro Plus 6.0 (Media Cybernetics, US).

### Statistical analysis

Each parameter was expressed as the mean ± SEM. Statistical significance was determined by t-Student’s test. A *p* value of less than 0.05 was considered significant.

## Results

### Sucrose consumption test

**Figure 1:**
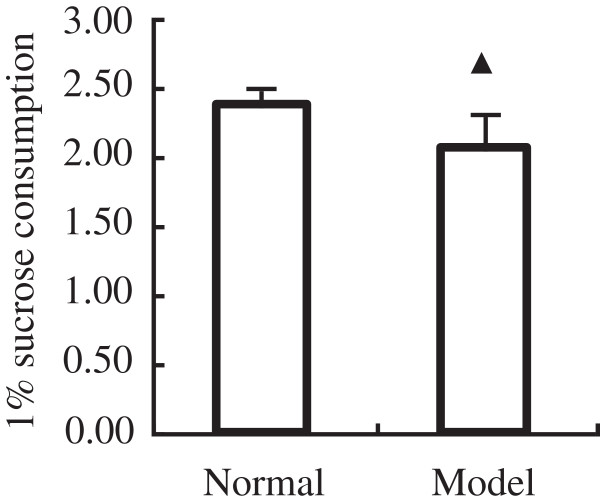
**Effects of a 14-day treat with tail clipping on 1% sucrose consumption.** The results represent the mean ± SEM of values obtained from 8 rats in each group. The significance of differences from the normal group at ^▲^*P*<0.05.

Compared with the activity of sucrose consumption of normal group, 2.36 ± 0.15, the activity of sucrose consumption of model group, 2.06 ± 0.25, is much lower than compared with the activity of sucrose consumption the normal group, which states notable statistic difference (*P*<0.05) (Figure 1).

### Acute stress exposure resulted in depression-like symptoms

**Figure 2:**
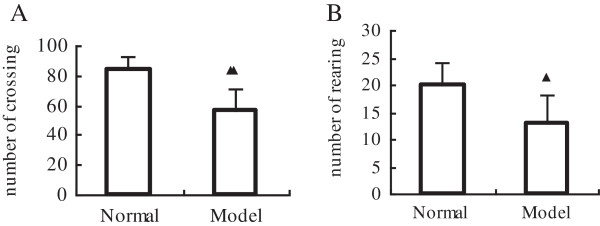
**Comparison of behavioral motor effects elicited by acute stress in open-field test. A**, Horizontal activity estimated as the number of grids crossed during a 3-minute test. **B**, The frequency of rearing with hind legs during a 3-minute test. The data were obtained one day before sacrificed. Data are expressed as mean ± SEM (n = 8). ^▲^*P*<0.05 and ^▲▲^*P*<0.01 compared with the normal group.

Compared to the activities of the horizontal and the vertical of normal group, which 84 ± 9 and 20 ± 4, the activities of the horizontal and the vertical of model group were much lower, the results of these two indexes were 56 ± 15 and 13 ± 5. We can find remarkable statistical difference (*P*<0.01 or *P*<0.05) (Figure 2).

### Body weight and liver coefficient

**Figure 3:**
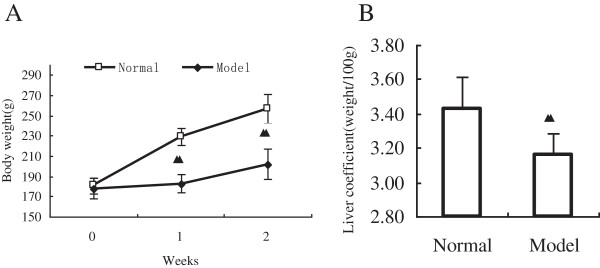
**Effect of acute stress on body weight and liver coefficient in rats. A**, Results of rat’s weight of submitted after 2 weeks of acute stress. **B**, Results of the liver coefficient of rats submitted after 2 weeks of acute stress. Data are expressed as mean ± SEM (n = 8). ^▲▲^*P*<0.01 versus the normal group.

As shown in Figure 3A, during the control phase (baseline), all animals (normal and model groups) exhibited a similar weight, 180.7 ± 8.1 g and 178.1 ± 10.0 g. However, after two weeks of acute stress, reduced this preference in the model group, which are 183.0 ± 8.6 g and 202.2 ± 14.5 g, the body weight of normal group in the next were 229.7 ± 8.5 g and 256.8 ± 14.7 g, the differences between two groups extremely remarkable difference (*P*<0.01) (Figure 3A). As shown in Figure 3B, two weeks of acute stress decrease the relative liver coefficient (weight/100 g body weight), 3.16 ± 0.12, the liver coefficient of normal group, 3.43 ± 0.18, is much higher as compared with the liver coefficient of model group, which states notable statistic difference (*P*<0.01) (Figure 3B).

### Comparison of serum and blood lipid metabolism index between each group

The level of TC, TG, LDL-C and HDL-C of the normal rats were 1.56 ± 0.14 mmol/L, 0.46 ± 0.14 mmol/L, 0.22 ± 0.03 mmol/L, 0.73 ± 0.06 mmol/L, comparing to the normal group, the rats have a higher level of TC, 1.61 ± 0.12 mmol/L, higher level of TG, 0.47 ± 0.14 mmol/L, but shown no statistical differences (*P*>0.05), a clear higher level of LDL-C, 0.30 ± 0.07 mmol/L, a lower level of HDL-C at 0.61 ± 0.05 mmol/L, which show remarkable statistic differences (*P* < 0.05 or *P* < 0.01) (Table 2).

**Table 2:** Comparison of serum lipid metabolism index between each group

	**Control(n = 8)**	**Model(n = 8)**	**T-test results**		
			**t**	**df**	**sig**
TC(mmol/L)	1.56 ± 0.14	1.61 ± 0.12	−0.841	14	0.415
TG(mmol/L)	0.46 ± 0.14	0.47 ± 0.14	−0.142	14	0.889
LDL(mmol/L)	0.22 ± 0.03	0.30 ± 0.07^▲^	−2.916	14	0.011
HDL(mmol/L)	0.73 ± 0.06	0.61 ± 0.05^▲▲^	4.57	14	0.000

Data are presented as mean ± SEM. ^▲^*P*<0.05 and ^▲▲^*P*<0.01 compared with the normal group.

### Comparison of serum liver function index between each group

Compared with the activities of ALB, GLB, ALT and AST of normal group, which are 38.9 ±0.8 g/L, 21.3 ± 1.1 g/L, 43 ± 12 U/L and 97 ± 15 U/L, respectively, the rats in model group has a lower level of ALB, 34.8 ± 2.8 g/L, a higher level of GLB, 23.0 ± 1.0 g/L, the difference between these two groups were statistically significant (*P* < 0.01). The level of ALT and AST of the rats in model group were separate at 41 ± 10 U/L and 105 ± 29 U/L, comparing to the index of normal group, it illustrate no statistical differences (*P*>0.05) (Table 3).

**Table 3:** Comparison of serum liver function index between each group

	**Control(n = 8)**	**Model(n = 8)**	**T-test results**		
			**t**	**df**	**sig**
ALB(g/L)	38.9 ± 0.8	34.8 ± 2.8^▲▲^	3.896	14	0.002
GLB(g/L)	21.3 ± 1.1	23.0 ± 1.0^▲▲^	−3.222	14	0.006
ALT(U/L)	43 ± 12	41 ± 10	0.33	14	0.745
AST(U/L)	97 ± 15	105 ± 29	−0.77	14	0.453

Data are presented as mean ± SEM. ^▲▲^*P*<0.01 compared with the normal group.

### Acute stress affects lipid metabolism in rats

**Figure 4:**
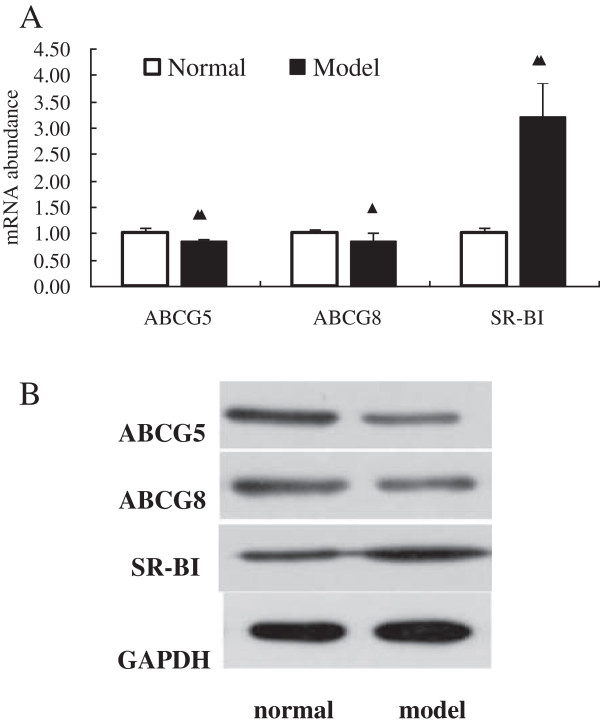
**Acute stress affects lipid metabolism was associated with the inhibition of ABCG5, ABCG8 and SR-BI. A**: The expression pattern of messenger RNA (mRNA) of ABCG5, ABCG8 and SR-BI in the liver measured with quantitative polymerase chain reaction. **B**: Western blots illustrate acute stress decreased ABCG5 and ABCG8, while increased SR-BI. Data are presented as mean ± SEM (n = 8). ^▲^*P*<0.05 and ^▲▲^*P*<0.01 versus the normal group, t-test, (ABCG5: t = 3.597; ABCG8: t = 2.61; SR-BI: t = −9.335).

We examined the effects of acute stress on expression levels of liver ABCG5, ABCG8 and SR-BI using western blotting. As shown in Figure 4B, the expressions of ABCG5 and ABCG8 of all of these livers were lower in model rats, while SR-BI was higher. These results were confirmed by quantitative PCR assays showing that messenger RNA (mRNA) levels of ABCG5 and ABCG8 were all down-regulated in model rats (Figure 4A) whereas SR-BI mRNA seemed to be up-regulated.

## Discussion

Anger is one of the seven emotions in Traditional Chinese medicine (TCM). Chinese medicine theory believes raged harms normal liver function, ‘raged impairing liver’. *SU WEN · The disease theory*, wrote “In great anger, an increase in *Qi* damages the liver.” Meanwhile, *SU WEN · Theory of Yin and Yang*, wrote “anger, as liver emotion”, the so-called anger is metaplasia by the vital essence. If anger hurt viscera, it hurts the born of the viscera.

Stress was put forward by Han Salye in 1944, it is a kind of syndrome reaction state including nerve, endocrinology and immunity, when the body faces strong stimulus from the outside world. When confronting stressors, the hypothalamic-pituitary-adrenal axis is excited during stress, the adrenal endocrine releases more glucocorticoids, meanwhile, the glucose level in blood is also higher, and the immunity function is depressed. Good stress reaction is beneficial to the body in a short term environment, if the body under stress for an extended time, the HPA axis is over excited, GC level is too high, then the body is hurt. Stress has been shown to play an important role in the onset of cardiovascular disease [17-20], it has also been found to inhibit metabolic functions such as lipid elimination and glycometabolism [5,6].

There have studies [8-11] reported that stress has some influences on the liver, such as oxidative stress. According to Chinese medicine, rage harms the liver, since liver plays a great role in lipid metabolism, studying the impact of acute stress on lipid metabolism helps to explain the theory. This study established a raged-rat model (refers to a method described in *Experiment of Zoology*, issued by Science Press, China) by clipping its tail, three times a day for 30 minutes each time. In the preliminary experiments, we found that the method mentioned in *Experiment of Zoology* caused huge damages to rats, we determined to adjust this tail clipping to three times a day. We found acute stress affected the behaviors of rats, which is proved in the open-field test, a method used to evaluate the behavior of animal. The horizontal scores of open-field test reflects the excitability of rats, while vertical scores reflects the rat’s to the environment [21,22]. Both the activities of the model group are lower than the normal group, the result shows excitability and exploration activities of rats decreased due to being subjected to stress.

With normal rats, we found serum ALB level was lower in the model group, with higher GLB, LDL-C and lower HDL-C, demonstrated acute stress may damage rat’s liver, leading to a reduction in ALB production, an increase in GLB. ALB is manufactured in the liver, when liver is impaired, it decreases the production of ALB. At the same time, GLB is manufactured by immune organ, when there is an ‘enemy’ in the body, GLB increase in production. Serum concentrations of HDL-C have strong inverse correlations with the risk of cardiovascular disease, independently of LDL-C levels, indicate that acute stress may influencing the HDL-C and LDL-C levels of serum, thus affecting the liver lipid metabolism. Due to the stress of time is not long or strong enough, the extent of the damage of liver cells is not particularly serious. So there are no significant differences in ALT and AST levels between the normal group and model group in the present study.

In this study, the liver tissue of model group exhibited a lower gene expression of ABCG5, ABCG8 and a higher SR-BI level, when compared to normal rats. Furthermore, liver protein expression of ABCG5 and ABCG8 were lower in the model group than the normal group, while SR-BI was higher in the model group than the normal group. ABCG5 and ABCG8 are ATP-binding cassette transporters, which expressed mainly in the liver and small intestine, mediated lipid membrane unidirectional outflow into the extracellular. After acute stress applied, ABCG5 and ABCG8 decreased expression in the liver, henced the intracellular lipid efflux was suppressed. The results show that, acute stress may affect the liver lipid efflux from the cells, thereby affecting the normal operation of the lipid metabolism, and may also be one of the reasons the Chinese say ‘raged impairing liver’. SR-BI is one kind of HDL receptors, first determined at the molecular level, having highly binding force with HDL, and mediated hepatic selective uptake of cholesterol ester. Increased the expression of liver SR-BIcan be effective in promoting reverse cholesterol transport. But in this study, SR-BI expression increased after acute stress was applied. This may be in the early acute stress process, mature HDL decreased in production, SR-BI expression in the liver cell membrane transient up-regulation in order to better capture of HDL. Specific mechanism needs to be followed up in future studies.

## Conclusions

In conclusion, we confirmed here for the first time acute stress decreased the expression of hepatic ABCG5 and ABCG8 besides up-regulated SR-BIin raged rats. Our data shown that acute stress harms normal liver functions, affects the level of lipid metabolism. Elevated serum GLB and LDL-C levels, reduced serum ALB and HDL-C levels, inhibited of reverse cholesterol transporter gene expression may all contribute to the harmful effects of acute stress. Our findings help to explain the Chinese medicine terminology of raged impairing liver.

## Abbreviations

ALT: Alanine aminotransferase; AST: Aspartate aminotransferase; ALB: Albumin; GLB: Globulin; TC: Total cholesterol; TG: Total triglyceride; HDL-C: High-density lipoprotein cholesterol; LDL-C: Low-density lipoprotein cholesterol; SR-BI: Scavenger receptor BI; ABCG: ATP-binding cassette transporter.

## Competing interests

The authors declare that they have no competing interests.

## Authors’ contributions

XLG participated in the design of the study, and performed statistical and genetic analysis and drafted the manuscript. SQL carried out by the Western blotting. YEZ performed blood samples analysis. SLW conceived of the study, and participated in its design and coordination and helped to draft the manuscript. All authors read and approved the final manuscript.

## References

[B1] Esch T Health in stress: change in the stress concept and its significance for prevention, health and life style Gesundheitswesen 2002 64 73 81 10.1055/s-2002-20275 11904846

[B2] Esch T Stefano GB Fricchione GL Benson H Stress in cardiovascular diseases Med Sci Monit 2002 8 93 101 12011786

[B3] Chen WW He RR Li YF Li SB Tsoi B Kurihara H Pharmacological studies on the anxiolytic effect of standardized *Schisandra lignans* extract on restraint-stressed mice Phytomedicine 2011 18 1144 1147 10.1016/j.phymed.2011.06.004 21757327

[B4] He RR Yao XS Li HY Dai Y Duan YH Li YF Kurihara H The anti-stress effects of *Sarcandra glabra* extract on restraint-evoked immunocompromise Biol. Pharm. Bull 2009 32 247 252 10.1248/bpb.32.247 19182384

[B5] Gopaul NK Manraj MD Hebe A Lee Kwai Yan S Johnston A Carrier MJ Anggard EE Oxidative stress could precede endothelial dysfunction and insulin resistance in Indian Mauritians with impaired glucose metabolism Diabetologia 2001 44 706 712 10.1007/s001250051679 11440363

[B6] Gigon A Matos AR Laffray D Zuily-Fodil Y Pham-Thi AT Effect of drought stress on lipid metabolism in the leaves of *Arabidopsis thaliana* Ann Bot 2004 94 345 351 10.1093/aob/mch150 15277243 PMC4242175

[B7] Gonzalez-Flecha B Cutrin JC Boveris A Time cource and mechanism of oxidative stress and tissue damage in rat liver subjected to in vivo ischemia-reperfusion J Clin Invest 1993 91 456 464 10.1172/JCI116223 8432855 PMC287955

[B8] Mittler R Oxidative stress, antioxidants and stress tolerance Trends Plant Sci 2002 7 405 410 10.1016/S1360-1385(02)02312-9 12234732

[B9] Berlett BS Stadman ER Protein oxidation in aging, disease, and oxidative stress J Biol Chem 1997 272 20313 20316 10.1074/jbc.272.33.20313 9252331

[B10] Pu HJ Cao YF He RR Zhao ZL Song JH Jiang B Huang T Tang SH Lu JM Kurihara H Correlation between Anti-stress and Hepatoprotective Effects of *Schisandra Lignans* Was Related with Its Anti-oxidative Actions in Liver Cells Evidence-Based Complementary and Alternative Med 2012 2012 161062 10.1155/2012/161062 PMC3385912 22792122

[B11] Zhai YJ He RR Tsoi B Li YF Li XD Tsuruoka N Abe K Kurihara H Protective effect of extract of chicken meat on restraint stress-induced liver damage in mice Food Function 2012 3 662 667 10.1039/c2fo10275g 22426689

[B12] Ye D Lammers B Zhao Y Meurs L Van Berkel TJ Van Eck M ATP-binding cassette transporters A1 and G1, HDL metabolism, cholesterol efflux, and inflammation: important targets for the treatment of atherosclerosis Current Drug Targets 2011 12 647 660 10.2174/138945011795378522 21039336

[B13] Hoekstra M Van Berkel TJ Van eck M Scavenger receptor BI: a multi-purpose player in cholesterol and steroid metabolism World J Gastroenterol 2010 16 5916 5924 21157967 10.3748/wjg.v16.i47.5916 PMC3007109

[B14] Fabre AC Malaval C Ben Addi A Verdier C Pons V Serhan N Lichtenstein L Combes G Huby T Briand F Collet X Nijstad N Tietge U Robaye B Perret B Boeynaems JM Martinez LO P2Y13 receptor is critical for reverse cholesterol transport Hepatology 2010 52 1477 1483 10.1002/hep.23897 20830789

[B15] Zou YH Xu ZW Su GQ Zou YH Animal models of human diseases: the experiment of zoology: chapter 7 2010 6 China: Science Press 140

[B16] Dunn AJ Swiergiel AH Effects of interleukin-1 and endotoxin in the forced swim and tail suspension tests in mice Pharmacol Biohem Behav 2005 81 688 693 10.1016/j.pbb.2005.04.019 PMC1975689 15982728

[B17] Bassenge E Schneider HT Daiber A Oxidative stress and cardiovascular diseases Dtsch Med Wochenschr 2005 130 2904 2909 10.1055/s-2005-923325 16342016

[B18] Nageswara RM Aleksandr V Marschall SR Oxidative stress and vascular disease Arterioscler Thromb Vasc Biol 2005 25 29 38 15539615 10.1161/01.ATV.0000150649.39934.13

[B19] Antelava NA Pachkoriia KZ Kezeli TD Nikuradze NS Shamkulashvili GG Major pathogenic links of atherosclerosis Georgian Med News 2005 128 72 79 16369071

[B20] Yokoyama M Oxidative stress and atherosclerosis Curr Opin Pharmacol 2004 4 110 115 10.1016/j.coph.2003.12.004 15063353

[B21] Wei W Wu XM Li YJ Ma Y, Zhou WX, Zhang YX Behavioral pharmacology experiment method: methodology of pharmacological experiment: chapter 19 2010 4 China: Pepole’s Medical Publishing House 635 636

[B22] Guo DY Chen TY Li B Zhang L Li YL Li L Behavioral comparison among the rats at different age in water maze and open field Acta Laboratorium Animalis Scientia Sinica 1998 6 19 23

